# Protocol for a randomized controlled trial evaluating the artificial intelligence health education accurately linking system in patients with mild-to-moderate stroke

**DOI:** 10.3389/fneur.2025.1737297

**Published:** 2026-01-07

**Authors:** Zhixia Liu, Yun-Hua Li, Yuan Fang, Huili Wang, Tao Wu, Shu Liu, Yanming Yang, Yangyang Qin, Xiaoge Tao, Jing Mao, Lijun Wang, Xiangmei Li, Xiaoyi Wang, Ruirui Yang, Yi Liu, Mengke Chen, Dandan Shi, Nan Li, Yajuan Wang, Yi Hu, Shumei Zhang

**Affiliations:** 1 Nursing Department, the First Affiliated Hospital of Henan University of Chinese Medicine, Zhengzhou, China; 2 School of Education, Chengdu College of Arts and Sciences, Chengdu, China; 3 School of Nursing, Henan University of Science and Technology, Luoyang, China; 4 The Brain Disease Regional Diagnosis and Treatment Center, the First Affiliated Hospital of Henan University of Chinese Medicine, Zhengzhou, China; 5 Department of Neurology, the First Affiliated Hospital of Henan University of Science and Technology, Luoyang, China; 6 Intensive Care Unit, the First Affiliated Hospital of Henan University of Science and Technology, Luoyang, China; 7 School of Nursing, Shandong Second Medical University, Weifan, China; 8 Nursing Department, the First Affiliated Hospital of Henan University of Science and Technology, Luoyang, China

**Keywords:** artificial intelligence, large language model, mobile health, randomized controlled trail, stroke

## Abstract

**Background:**

Stroke is a leading cause of death and disability worldwide. Although survival rates from mild-to-moderate stroke are high, long-term functional impairment remains common, requiring sustained self-management beyond traditional rehabilitation. Conventional models depend on institutional medical care, which not only drives up costs but also disrupts continuity of care. Meanwhile, psychological, risk-related, and behavioral factors are often overlooked. Advances in artificial intelligence (AI) and mobile health provide opportunities for individualized, long-term support. Based on this, we developed the AI Health Education Accurately Linking System (AI-HEALS) to evaluate its potential to improve physiological parameters, risk perception, and self-management in patients with mild-to-moderate stroke.

**Methods:**

This single-blind randomized controlled trial evaluates AI-HEALS, delivered via WeChat (China’s most widely used social media app), to improve the monitoring of key physiological indicators in patients with mild-to-moderate stroke. Eligible participants are randomly allocated either standard care as a control or standard care plus a three-month regimen of AI-HEALS. It features an AI-powered interactive Q&A system that supports both voice and text communication, real-time monitoring of physiological and behavioral indicators, personalized health reminders, and specially designed educational content. These are all offered through the official WeChat account “Stroke Health Management Expert.” The primary outcomes are changes in blood pressure, glucose, and blood lipids. Secondary outcomes include risk perception of recurrence of stroke, self-management behaviors, and psychological state of mind. Follow-up assessments are conducted at 3, 6, and 9 months after completion of the intervention to evaluate both short-term and sustained effects.

**Discussion:**

This protocol presents a new AI-mHealth approach to delivering stroke care. If proven feasible and effective, AI-HEALS could offer a scalable and sustainable model for improving long-term health outcomes, reducing the risk of recurrence, and optimizing the use of healthcare resources for stroke and other chronic conditions.

**Clinical trial registration:**

https://www.chictr.org.cn/showproj.html?proj=251515, Identifier, ChiCTR2500096422.

## 1 Introduction

Stroke is one of the leading causes of death as well as disability on a worldwide basis, endangering human health severely (1). According to the Global Burden of Disease Study 2019, stroke accounted for approximately 6.55 million deaths worldwide, with 101 million people living with the condition and a total of 143 million disability-adjusted life years (DALYs) lost (2). The incidence of stroke rises sharply with population aging, a trend that is particularly pronounced in China (3). Stroke has both a high incidence as well as deep impact on patients’ lives of daily living as well as occupational functioning (4). Speech dysfunction, movement, and cognition, are easily affected by stroke, causing evident quality of life decrement (4, 5). Moreover, stroke puts a tremendous economic burden on patients’ families, as long-term care and rehabilitation requires them financially for years at a stretch (2, 6). Caregivers themselves may develop psychological disturbance and physical disorders (7, 8). Medical expenses and societal expenses of stroke are enormous (6). According to the American Heart Association, stroke imposes a substantial economic burden on the United States health system, costing approximately $34 billion each year (9). As population aging keeps accelerating, incidence of stroke as well as related burdens keep rising (10).

Current standard rehabilitation for patients with mild-to-moderate stroke typically includes physical therapy, occupational therapy, speech therapy, and structured health education (11, 12). These approaches mainly target the restoration of physical functioning, improvement in activities of daily living, and facilitation of community reintegration. These are primarily focused on assisting and supporting patients in restoring their physical functions and helping them to return to society. Additionally, new concepts, such as virtual reality therapy and therapy assisted by robots, are adding new dimensions to the available options for patients (13), while there are some limitations to the present model. First, traditional rehabilitation generally requires patients to attend specialized facilities in person. This requirement can impose substantial financial and logistical burdens, particularly with respect to travel, time commitments, and repeated appointments (14). These difficulties tend to limit the frequency and ease of rehabilitation, which are important for optimal recovery. Second, the standard approaches to rehabilitation are primarily physical and do not emphasize psychological well-being, the perception of risk, and behavioral shifts as much. However, psychological distress, lack of confidence, and ineffective behavior shifts are well-established poor predictors of secondary prevention and the quality of life for survivors of stroke (15). The standard care does not contain inherent components that address such aspects. Finally, while technology-assisted or remotely delivered rehabilitation, such as videoconferencing-based assessments and home-based telerehabilitation, has shown promising functional and neural outcomes (16, 17), these approaches remain primarily oriented toward motor recovery rather than comprehensive self-management support. They often do not receive continuous health education, behavioral support, and combined tracking of health and lifestyle factors. As such, despite advanced technology, the present care model fails to comprehensively address the needs for managing patients with mild to moderate stroke.

Mobile health (mHealth) and artificial intelligence (AI) technologies have simplified personalized and continuous support for people living with chronic conditions. mHealth applications provide distant access to health information, reminders, and patient self-check facilities. AI can improve such applications by allowing for response generation, tailored content, and proper use of patient-submitted information. These concepts assist patients to stay engaged and simplify their overall self-management. For the purpose, the Artificial Intelligence Health Education Accurately Linking System (AI-HEALS) was designed as a WeChat-based tool for complementing the conventional rehabilitation process for patients with mild to moderate stroke. AI-HEALS incorporates proven behavioral model concepts, the Health Action Process Approach (HAPA) and the Multi-Theory Model (MTM) (18, 19), to direct the design of the education and behavioral components. AI-HEALS consists of the following functional elements: an AI-supported question-and-answer section with an organized and updated knowledge base, the capacity for the recording of healthcare and behavioral information, reminders, and health education. Though named AI-HEALS, the system actually serves as an AI-driven health education and management system and not as therapy. This tool would assist patients in managing their health outside the healthcare environment.

Based on it, this protocol describes a single-blind, randomized controlled trial (RCT) designed to systematically evaluate the effectiveness of the AI-HEALS in promoting self-health management among patients with mild to moderate stroke. The specific objectives of the study are:

- To determine whether a 3-month AI-HEALS–based comprehensive intervention improves physiological indicators, including blood pressure, blood glucose, and body weight;
- To assess the intervention’s impact on psychological outcomes, such as anxiety, depression, and self-efficacy;
- To evaluate the effectiveness of the intervention in enhancing self-management behaviors, including medication adherence, engagement in rehabilitation exercises, and healthy dietary practices.

## 2 Methods

### 2.1 Study design

**Figure 1:**
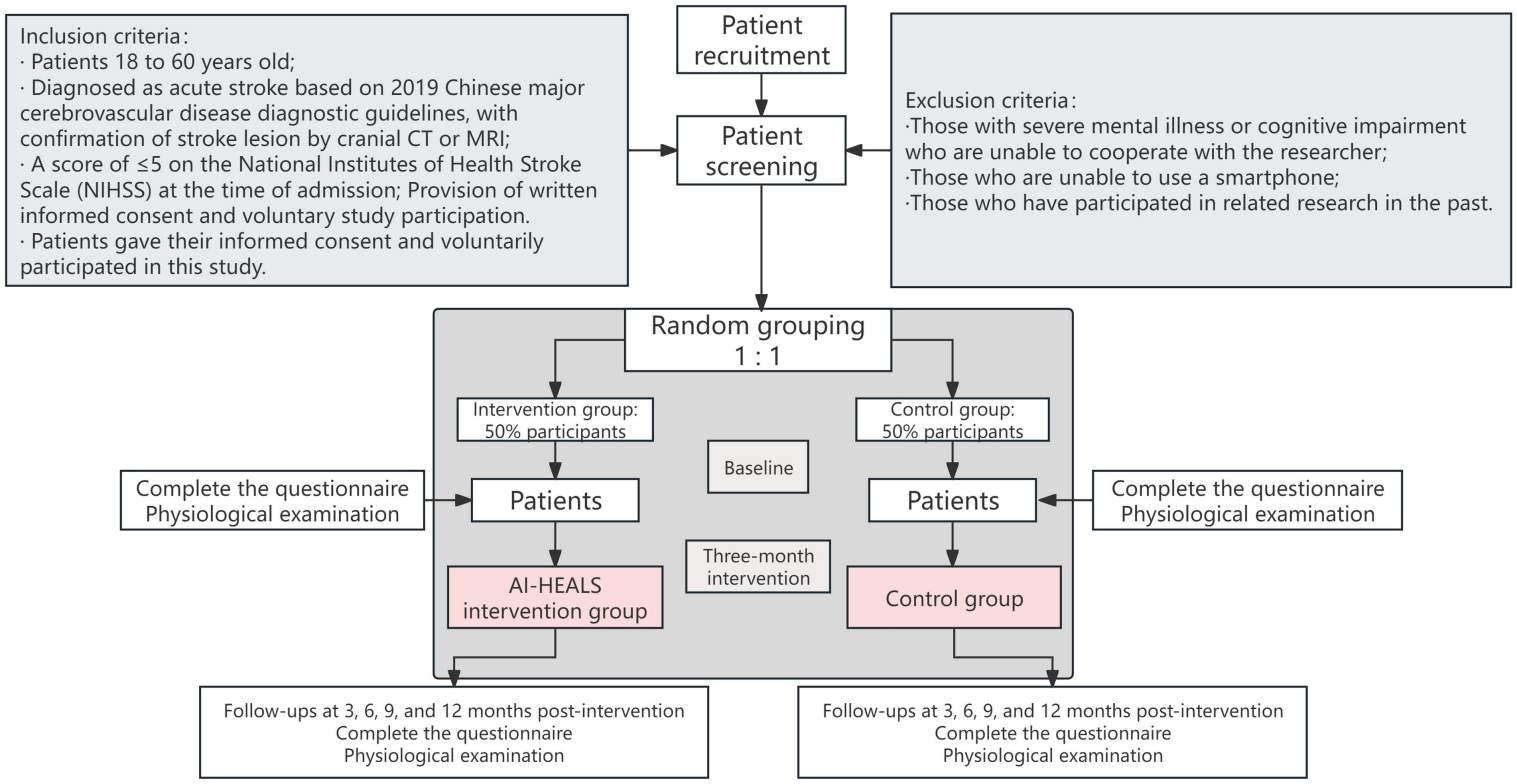
Flow chart of patient recruitment and study implementation.

The research design of our study will be a single-center, randomized, single-blind, parallel-group clinical trial. The subjects of the study will be patients with mild to moderate stroke from the public general hospital of Luoyang, China. Figure 1 shows the complete workflow of the study as well as group allocation procedures.

### 2.2 Randomization and blinding

Single-blinding will be applied for the study, because the AI-HEALS intervention would not be possible for the participants and the intervention team to be blinded regarding the allocation of the groups. In the context, single-blinding indicates that the people involved in the evaluation process and analysis are blinded to the allocation groups of the participants during the evaluation and data analyses. For the randomization process, it would implement the use of the computer-generated random number sequence. For allocation, it would use the 1:1 allocation ratio. An independent team member who is not involved in participant recruitment, clinical care, or intervention delivery will generate the randomization sequence and manage allocation concealment. Group assignments will be released to the intervention team only after allocation is complete to ensure that recruitment personnel remain blind to group assignment at the time of enrollment. For avoiding sharing between allocation groups, the participants would be admitted to separate units according to their allocation (Ward A for the intervention and Ward B for the control groups). Although participants and care providers cannot be blinded, several measures will be implemented to reduce performance and detection bias:

- Outcome data will be collected by research assistants who are blinded to group allocation.
- Data analysis will be performed by statisticians who are blinded to group identities, with datasets fully de-identified prior to analysis.
- Randomization and allocation procedures will be managed by an independent researcher not involved in recruitment or intervention provision.
- Physical separation of inpatient units will minimize interaction between participants from different groups.

All data will remain securely stored and de-identified to maintain the integrity of the blinding process.

### 2.3 Study sample

Consecutive sampling method will enroll stroke patients currently receiving treatment at a tertiary Grade A hospital of Luoyang, China, from February 2025 to February 2026. Inclusion and exclusion criteria are set as follows:

Inclusion Criteria: Patients 18 to 60 years old; Diagnosed as acute stroke based on 2019 Chinese major cerebrovascular disease diagnostic guidelines (20), with confirmation of stroke lesion by cranial CT or MRI; A score of ≤5 on the National Institutes of Health Stroke Scale (NIHSS) at the time of admission (21, 22); Patients gave their informed consent and voluntarily participated in this study.

Exclusion Criteria: Those with severe mental illness or cognitive impairment who are unable to cooperate with the researcher; those who are unable to use a smartphone; and those who have participated in related research in the past.

Withdrawal Criteria: Voluntary drop-out of the participant due to personal reasons (e.g., scheduling, loss of interest); During the research, seriousness of severe adverse events, such as severe illness or acute illness episode (e.g., myocardial infarction, serious infection), that requires the cessation of the intervention; Clinical changes or new guidelines for treatment that would necessitate alterations to the participant’s treatment regimen that may conflict with the study intervention.

### 2.4 Sample size

This research study was designed as a single-blind RCT. The primary objective of the study was to ensure if the AI-HEALS intervention program is feasible, acceptable, and easily implementable, as opposed to determining the efficacy of the program. Thus, it is sample size was determined based on the format for conducting a pilot study as proposed by Billingham et al. (23). Based on such recommendations, the minimum number of participants for our study would be 50, consisting of 25 per group, to ensure the objectives are achieved for the pilot study. Taking into consideration the expected dropout of 20%, the minimum number of people to recruit would be 60, 30 for each group. This sample size is expected to provide reliable estimates of feasibility parameters and preliminary effect size estimates for planning a subsequent fully powered RCT.

Although the pilot trial is not designed to have statistical power for testing hypotheses, it will provide an estimate of the average and variability in the primary outcome variables, such as blood pressure, glucose, and lipid levels. These estimates of effect size would then help design the study size for the future larger randomized clinical trial, which would be powered for testing the intervention at the conventional levels of alpha = 0.05 and 80–90% power.

### 2.5 Recruitment

This study shall enroll patients with mild to moderate stroke as outlined by the inclusion and exclusion criterion set, to evaluate the efficacy of the AI-HEALS intervention. Recruitment shall be done by the hospital clinical team, which shall screen potential participants upon admission and provide them with the option of enrolling into the study. Subsequently, the study team shall conduct face-to-face consultations, which shall involve clarifying the study goals, procedures, potential benefits, and associated risks. Eligible patients shall be asked to receive a thorough health evaluation, involving discussion of the history of their stroke and current symptom. Only when there shall be complete understanding of the study, including clarification of all questions, shall patients be asked to provide an informed consent form. The participants shall only enroll into the study upon signing of the written informed consent form. Over the three-month AI-HEALS intervention, assessments will be conducted at baseline (pre-intervention), at discharge, at the end of the intervention, and during follow-up. To encourage maximum patient participation, all participants will receive a free medical consultation at the end of the study as an incentive for their participation.

### 2.6 Informed consent

Informed consent process shall be carried out openly, transparently, strictly abiding by ethic values. Patients meeting inclusion, exclusion criteria shall be given a clear, detailed, written statement of the primary objectives of the study, special procedures, potential risks as well as benefits, including data protection available to them. It shall include all explanations of how their data shall be collected, stored, secured. All data shall be saved on secure, encrypted format on specially guarded servers, readable only by authorized personnel of the research team once they are on the study, disposed of properly at study completion.

Participants will have sufficient time to read the informed consent form, pose questions, and willingly offer their written consent. They will fully be made aware of the privilege of withdrawing from the study at whatever time without adverse effects on them. The research team will offer sustained contact details to ensure that participants may, at whatever time, request further clarification or help. During the study, new information that may influence participants’ motivation to continue will, at once, be revealed, hence preserving ongoing informed consent throughout the study.

Moreover, participants will incur no penalty for choosing to withdraw from the intervention group. If the intervention has already begun, patients choosing to withdraw will never be transferred into the control group but are retained as dropouts. Semi-structured interviews, as far as possible, shall be conducted on such persons regarding reasons for terminating. During consent, participants shall be specifically informed of all post-withdrawal decisions such that at all steps they remain fully aware of decisions as well as rights of theirs that persist over time of study.

### 2.7 Intervention

#### 2.7.1 Control group: standard care group

The participants assigned to the control arm shall receive standard care offered by a multidisciplinary clinical team. Such standard care shall include the following: Regular visits to physicians for ongoing assessment of well-being and refinement of treatment; Scheduled follow-up visits to evaluate disease progression; Systematic diagnostic tests to screen selected health indicators; Carried out structured education on health on managing diseases, treatment adherence, and lifestyle changes.

#### 2.7.2 Intervention group: standard care + AI-HEALS

Participants of the intervention group shall receive standard care and a three-month AI-HEALS intervention from the WeChat service platform “Stroke Health Management Expert.” The intervention team is composed of public health professionals, physicians, clinical nurses, psychologists, and statisticians, each contributing their specialized expertise to the design and implementation of the intervention. The program consists of the following four core program elements:

- Knowledge-Based Question Answering System on Stroke (KBQA): This module would enable patient education on stroke issues through an intelligent FAQ chatbot (FAQRobot), such that patients obtain accurate and efficient knowledge on stroke. To achieve the same, a deep and credible knowledge base shall be built, covering a wide topic spectrum including basic concepts, etiology, symptom, treatment, medication regimen, day-to-day control measures, healthy diet, and proper exercise. The knowledge base shall be incorporated into the Doubao Large Language Model (developed at ByteDance), such that the system would provide accurate, professional answers to patient questions fed through the WeChat messaging channel. To enhance interactivity and user engagement, the system will suggest three related follow-up questions based on each user query, encouraging patients to explore relevant content in greater depth. Voice-interaction capability is also provided, allowing patients to ask stroke-related questions through voice input. To continuously optimize the knowledge base and make user experience richer, the system would record patient questions and usage behavior (e.g., frequency of visits, time spent on the site) through backend monitoring, which would be analyzed to understand user behavior patterns. Such data shall further influence updating of knowledge base and question-answering system on a continuous basis, ensuring that the knowledge provided remains relevant, accurate, and up-to-date.
- Recording and Monitoring of Physiological Indicators and Lifestyle Changes: Participants will be asked to regularly record some of their lifestyle-linked data through the AI-HEALS system, e.g., food patterns, exercise, and medication adherence. These data will be entered into electronic form and uploaded into the system, which will allow research staff at all times to observe continuously patients’ self-management activities over time as well as changes over time for every patient. Such data analysis, and data collection, will make possible not only evaluation of the impact of the intervention on patients’ day-to-day activities but also optimization of person-centered treatment plans, thereby enhancing the overall effectiveness of health management.
- Personalized Reminder Services: To support treatment compliance, the AI-HEALS system provides customizable reminder functions. Patients can voluntarily set personalized alarms and the system will deliver reminders only at the times selected by the user. For example, medication schedules or monitoring tasks importantly. Additionally, participants may adjust the frequency of reminders or disable them at any time within the system, ensuring that the feature remains fully user-controlled and minimizes unnecessary burden.
- Automated and Personalized Stroke Self-Management Information: This study incorporates a kind of automated provision of information system through which participants will receive at the same time 1 to 3 informative messages each week on stroke self-management. The contents of such messages are proper diet suggestions, exercise guidelines, as well as medication concerns. What the researchers envision is patients’ health literacy improvement and people’s promotion toward utilizing enhanced self-care practices on a day-to-day basis. Additionally, the system through participants’ interacting chatbot data will intelligently provide sharper educative content that relates to particular needs. In order to optimize even further the outcomes of provision of information, the researchers at their side continuously evaluate backend data, including the number of articles, article types, and article reading time, each participant has progressed through. Relating to such outcomes, the content and provision frequency of the system will continuously change to make them more relevant, accurate, as well as efficient.

The “Doubao Pro-32 k” large language model (LLM) used here has been a Chinese-trained transformer-based model specifically optimized for uses of medical knowledge. It has received training on a combination of biomedical literature along with stroke-specific clinical guidelines and fine-tuned on a 32 k context window to facilitate the production of long-form health education content. Prior to domain-specific fine-tuning, pretraining of the model had covered extensively general language understanding to support its capabilities of performing medical reasoning. It had its performance evaluated on the third-party FlagEval evaluation platform,^1^ on which it demonstrated competitive performance against GPT-4.0. Doubao Pro-32 k had achieved an aggregate score of 77.75, significantly outstripping GPT-4.0’s 73.51 score. Most importantly, its best performance was on the domain of knowledge application, where it achieved 91.14, as opposed to GPT-4.0’s 86.71—a particularly crucial competency to create evidence-based clinical information. Following development, the model’s output had garnered initial expert assessment to make it aligned with prevailing knowledge of medicine and meet rigorous requirements needed for purposes of clinical research studies. Furthermore, at the close of the pilot RCT, a continuous validation process shall be established. As part of the process, two experts shall, on a monthly basis, assess 10% of the model’s output through assessing its appropriateness and ensure the system’s sustained reliability as well as integrity.

Finally, protecting participant privacy and ensuring data security during interactions with the AI-HEALS system are priorities. All patient information gathered for the research study will be kept confidential and encrypted, and no personally identifiable information would be submitted to WeChat. Participants are free to withdraw their participation from the research study for any reasons they prefer and without penalty. In order for AI-produced content to be utilized correctly, the knowledge base of the system and the accuracy of the AI responses are carefully checked and revised by healthcare professionals. This ensures that the information produced by AI remains valid and that research study participants are not exposed to incorrect information.

### 2.8 Strategies to enhance intervention adherence

To promote participant adherence to the intervention, the study will deploy the following measures:

- Explicit Communication of Responsibilities: Responsibilities of participants over the duration of the period of observation would be clearly explained at the recruitment stage as well as at the time of informed consent. Attempt would be made to develop good rapport between participants and researchers such that there would be mutual commitment and trust.
- Comprehensive education and counseling: Throughout the study, participants will receive integrated education and counseling covering disease management, lifestyle modification, and the prevention of potential complications.
- Weekly System Use Observation: The research team shall extensively monitor participants from the intervention arm on a weekly basis on their use of the AI-HEALS system. It would keep participants continuously involved and serve to identify potential issues or poor points of participation early. Appropriate corrective action would be instituted as needed to continue ensure that there is involvement as well as adherence.

### 2.9 Outcomes

#### 2.9.1 Primary outcomes

The primary outcomes of this trial are changes in key physiological parameters among patients with mild-to-moderate stroke. These include: Blood pressure, Blood glucose, Blood lipid levels. Measurements will be collected at baseline, discharge, the end of the 3-month intervention, and at 3, 6, and 9 months post-intervention. These indicators constitute the primary endpoints as they represent the main clinical effects targeted by the AI-HEALS intervention.

#### 2.9.2 Secondary outcomes

##### 2.9.2.1 Self-management behaviors

Changes in self-management behaviors will be assessed across multiple domains, including: behavioral decision-making, nutrition preference, physical activity, medication adherence, smoking and alcohol control, sleep patterns, quality of life, functional autonomy in daily living.

##### 2.9.2.2 Psychosocial and cognitive measures

These include: perceived recurrence/stroke risk, health literacy, self-efficacy, depression, anxiety, stress, perceived social support. These outcomes evaluate psychological and behavioral dimensions associated with secondary stroke prevention.

#### 2.9.3 Exploratory outcomes

Exploratory outcomes reflect additional clinical, behavioral, and process-related indicators. These include Physiological monitoring indicators: level of consciousness, respiratory rate, heart rate and rhythm, height, weight, waist circumference, body mass index (BMI). Healthcare utilization indicators: 30-day hospital readmission and duration of hospital stay. Process indicators: recruitment and attrition rates, reasons for withdrawal, and differential characteristics between participants who discontinue the study and those who complete it.

### 2.10 Variable measurement

Sociodemographic measures will be obtained at baseline. Physical examinations and questionnaire evaluation are scheduled at the following time points: baseline, at hospital discharge, at the end of the intervention, and at 3, 6, and 9 months post-intervention (Table 1).

**Table 1:** The schedule of enrolment, interventions, and assessments.

Study period	Recruitment	Intervention		Follow-up		
Timepoint	0	Discharge	3 Months	3 Months	6 Months	9 Months
Eligibility screen	✓					
Informed consent	✓					
Allocation	✓					
Sociodemographic variables	✓					
RRPS-SP	✓	✓	✓	✓	✓	✓
BDMS-SP	✓	✓	✓	✓	✓	✓
PHQ4	✓	✓	✓	✓	✓	✓
PSSS-SF	✓	✓	✓	✓	✓	✓
NGSES-SF	✓	✓	✓	✓	✓	✓
EQ-5D-5L	✓		✓	✓	✓	✓
Smoking	✓		✓	✓	✓	✓
Alcohol consumption	✓		✓	✓	✓	✓
B-PSQI	✓		✓	✓	✓	✓
FCS-SF	✓					
eHEALS	✓					

BDMS-SP, the Behavioral Decision-Making Scale for Stroke Patients; B-PSQI, The new Brief Version of the Pittsburgh Sleep Quality Index-Short Form; eHEALS, The eHealth Literacy Scale; FCS-SF, The Family Communication Scale-Short Form; EQ-5D-5L, EuroQol-5 Dimensions 5 Levels; PHQ-4, the abbreviated Patient Health Questionnaire-4; PSSS-SF, The Perceived Social Support Scale; NGSES-SF, The New General Self-Efficacy Scale-Short Form; RRPS-SP, Recurrence Risk Perception Scale for Patients with Stroke.

#### 2.10.1 Sociodemographic variables

A specially prepared demographic questionnaire will be used to collect data on the following variables: sex, age, marital status, household registration, average number of persons per household, education, occupation, income, symptom of stroke, and detailed history of diseases.

#### 2.10.2 Anthropometric variables

Height, weight, waist circumference, and blood pressure of each participant shall once be measured through certified equipment for accurate measurements. Height and weight shall once be obtained through duly calibrated and certified electronic weighing scales. BMI shall once be calculated as weight in kilograms divided by the square of the height in meters. Waist circumference once shall once be obtained at umbilical level through the use of a measuring tape. Blood pressure, including both systolic and diastolic measurements, will be obtained once using a validated automated sphygmomanometer (24). Venous blood samples were collected during hospitalization and at follow-up visits. Glycated hemoglobin (HbA1c) was measured to reflect the average blood glucose concentration over the preceding 2–3 months, providing an indicator of long-term glycemic control (25). Lipid profiles included total cholesterol (TC), triglycerides (TG), high-density lipoprotein cholesterol (HDL-C), and low-density lipoprotein cholesterol (LDL-C) (26). Serum TC and TG were measured using enzymatic methods, which employ specific enzyme reactions to convert target lipid components into detectable products, offering high sensitivity and specificity (26). Serum HDL-C and LDL-C were determined using homogeneous assays, in which specific chemical reactions uniformly disperse lipoproteins to enable quantification of cholesterol in each fraction (26).

#### 2.10.3 Post-discharge engagement

Regular follow-up upon hospital discharge shall continue through WeChat communication, phone calls, or mobile texting. These contacts serve to keep participants continually informed and involved. Participants shall, at all times, receive prompt reports on the progress of the study, resolution of issues that arise, as well as scheduling of forthcoming follow-up assessments.

Standardized data entry of such research work shall be accomplished through an electronic data capture (EDC) system. A double entry system shall be utilized by two independent operators for validation, followed by logical checks of consistency of all raw data. The sensitive data shall be secured through encryption under the Unicode encoding scheme. The data shall be stored on password-protected servers, which implement role-based security that only allows authorized investigators to look at de-identified data. Additionally, a real-time backup system shall be put into action that transfers data to offsite cloud storage to ensure data integrity as well as facilitate disaster recovery.

Once participants’ data has been collected, all personally identifying information will be promptly removed to ensure confidentiality. This means that personal identifiers will be replaced by participant-specific codes. Only a limited number of authorized team members will obtain the linkage key that connects such codes to actual identities. The key will be kept separately at a highly secure site.

Under no circumstances will any personal data be disclosed to third parties without the participant’s explicit informed consent. Every member of the research team must also sign confidentiality agreements to commit them to utmost data privacy standards. From the time of study initiation to study completion, and even after study closure personal data of all types shall at all times remain safely stored on dedicated servers, which shall only be opened to authorized personnel only.

#### 2.10.4 Questionnaire assessments

Recurrence Risk Perception Scale for Patients with Stroke, created by Lin Beilei et al. in 2021, will be used to assess patients’ perceived stroke recurrence risk (27). It is well suited for the population of interest of the current study. Although the original scale comprises two parts, only the second part will be used in this study. The first part, a visual analog scale, has neither established reliability nor validity and does not contribute to the total score. The second part consists of 17 items, spread over three dimensions: perceived risk of disease-related factors (4 items), perceived risk of behavioral factors (6 items), and perceived severity of recurrence (7 items). Each item has a 3-point Likert scale, from “Disagree” through “Agree,” which has a score of 1 to 3, respectively, with total scores of 17 to 51, respectively. The greater the score, the larger the perceived recurrence risk. The Cronbach’s *α* of the entire section is 0.850, of the three sections, 0.875, 0.815, and 0.804, respectively.

For the assessment of behavioral decision-making of patients suffering from mild stroke, the study will employ the Behavioral Decision-Making Scale for Stroke Patients, which has also been created by Lin Beilei et al. during 2022 (28). It has 30 items, spread over four dimensions: motivation for change of behavior (10 items), behavioral change intention (9 items), decision-making determinants (5 items), and decisional balance (6 items). It uses a 5-point Likert scale, on which the answers range from “Strongly Disagree” to “Strongly Agree,” on a 1–5 scale score. Its total score falls on a 30–150 scale, on which the larger values indicate larger behavioral decision-making capacity. Content validity of individual item falls on 0.800–1.000, while the total content validity falls on 0.975. Overall Cronbach’s α of the scale falls on 0.934, while the subscales record high internal consistency on 0.937, 0.933, 0.778, and 0.908, respectively.

Measurement of anxiety and depression will be conducted using the Patient Health Questionnaire-4 (PHQ-4), a brief self-report questionnaire designed to gage symptom presence over the past fortnight (29). The PHQ-4 includes four questions: the first two assess depressive symptom presence, and the final two assess anxiety symptom presence. Each question uses 4-point Likert scales, from “Not at all” (0 points) through to “Nearly every day” (3 points), such that the score range of the total lies between 0 to 12 points. The PHQ-4 has demonstrated notable validity and reliability on ethnically diverse populations, one validation study reporting 0.833 as Cronbach’s alpha of the Chinese version.

Perceived social support will be measured by the Perceived Social Support Scale–Short Form (PSSS-SF), which evaluates the perceived level of support of a person’s social network (30). Three items make up the scale, of which each one captures one of three specific types of support: family (item 1), friends (item 2), and other sources of support (item 3). Items are rated on a 7-point Likert scale from 1 (“Strongly disagree”) through 7 (“Strongly agree”) with a possible total score of 3 to 21. High values of the score indicate greater perceived available social support as well as the efficacy of the individual’s social network in supplying practical as well as affective support.

Participants’ self-efficacy as perceived ability to perform tasks or cope with demands will be assessed through the New General Self-Efficacy Scale–Short Form (NGSES-SF) (31). The three-item scale examines key self-efficacy facets: perceived ability (item 1), strength of confidence (item 2), and generality of confidence (item 3). Items use a 5-point Likert scale from 1 (“Strongly disagree”) through 5 (“Strongly agree”). Sum ratings of NGSES-SF total score range from 3 through 15. Higher ratings indicate greater self-confidence as well as a stronger perceived ability to cope with diversified demands.

The quality of life will be measured by the EuroQol-5 Dimensions 5 Levels (EQ-5D-5L) questionnaire. It is a well-validated instrument that measures five significant domains of health: mobility, self-care, usual activities, pain/discomfort, and anxiety/depression (32). Five response scales are provided for each of the five domains, from “no problems” to “extreme problems,” which allows respondents to select the best answer that reflects their current state of health at the moment of answering. These responses are used to obtain a complete health profile. In addition to the descriptive system, the EQ-5D-5L questionnaire includes a Visual Analog Scale (VAS), which permits respondents to self-rate the perceived current overall health on a 0-to-100 scale, from worst imaginable health to best imaginable health, providing a quantitative estimate of subjective well-being.

The smoking habit will be assessed by a specially prepared questionnaire consisting of four single-choice questions which attempt to capture participants’ smoking experience (33). The questions touch upon smoking history, years of abstinence (if ever smoked), cigarettes smoked daily, and initiation of smoking at what age. The first question has: “Do you currently smoke?” The options are: (1) Yes, traditional cigarettes; (2) Yes, electronic cigarettes; (3) Yes, both; (4) Used to smoke (stopped now); (5) Never smoked. Participants would be grouped into either current smokers or non-smokers (representing both never smokers and former smokers) based on these options.

Alcohol use assessment will also be conducted by a self-designed questionnaire consisting of seven single-choice questions seeking a total picture of participants’ consumption behavior (34, 35). The questionnaire includes whether the participant consumes alcohol, initiation and quit ages, alcoholic beverages consumed, consumption quantity per day, alcoholic beverages consumed before quitting, as well as quit-induced anxiety symptoms. Participants will respond to the question: “Do you currently or have you previously consumed alcohol?” as follows: (1) Never consumed alcohol; (2) Currently consumes alcohol; (3) Used to consume alcohol, but no longer does; (4) Did not consume alcohol previously, but currently does.

Sleep quality will be assessed through the use of the Pittsburgh Sleep Quality Index–Short Form (PSQI-SF), which is a shortened version of the initial PSQI, designed to evaluate the sleep quality of individuals (36). The PSQI-SF consists of six items from five core domains: sleep efficiency (items 1, 2), sleep latency (item 3), sleep duration (item 4), sleep disturbances (item 5), and subjective sleep quality (item 6). Wake time and bed time data, when available, are used for the calculation of sleep efficiency. The total score on the PSQI-SF ranges from 0 to 15, where elevated values reflect poor sleep quality and greater sleep issues.

The family communication will also be assessed by the Family Communication Scale–Short Form (FCS-SF), which identifies the quality of communication among family members (37). It consists of a four-item scale that captures one dimension of family communication, which is scored on a 5-point Likert scale from 1 (“Stronglydisagree”) through 5 (“Strongly agree”). The score varies from 4 to 20, of which greater values indicate more effective and healthier styles of communication among members of the family unit.

The Chinese version of the Electronic Health Literacy Scale (eHEALS) measures patients’ ability to search for, evaluate, and use electronic health information to solve health problems (38). The scale has five items on one dimension. It uses a 5-point Likert scale, where 1 indicates “strongly disagree” and 5 indicates “strongly agree.” The total score ranges from 5 to 25 points. Higher scores indicate better electronic health literacy.

### 2.11 Statistical analysis

Quantitative data will be gathered using structured questionnaires and would then be entered into Microsoft Excel for analysis. All major analyses would follow the intention-to-treat (ITT), and the per-protocol (PP) analysis would be performed for sensitivity testing. Descriptive statistics would be utilized for summarizing starting information, feasibility variables such as the number of participants, and use of the system.

For continuous data, the mean and standard deviation for normally distributed data, and the median and 25th to 75th percentiles for non-parametric data, will be presented. For comparison of groups initially untreated, and for groups measured once, independent samples t-tests and their non-parametric analogs would be employed. Categorical data would be expressed as frequency and percentage and would be analyzed using chi-squared tests. Normality of the data would be tested using the Kolmogorov Smirnov test.

Missing data will be handled using established multiple imputation methods where appropriate, under the assumption that data are missing at random. Patterns of missingness and attrition will be examined and compared between groups to identify potential differential dropout, and sensitivity analyses will be performed to compare imputed and complete-case results.

For the main trial, the focus would be on the use of the linear mixed-effects models for the evaluation of the primary outcome measures, such as the blood pressure, blood sugar, and blood fats. Additionally, the age factor would be incorporated as the covariate, which would account for the differences that people of varied ages would present, especially between the ages of 18 and 60. For secondary outcomes, such as the self-management and the psychosocial/cognitive outcomes, the analyses would use similar mixed-effects models or simpler comparative methods, depending on the nature and timing of the data collection. Also, exploratory age-stratified analyses may be conducted to descriptively examine potential heterogeneity of intervention effects across age groups, with results interpreted cautiously given the pilot nature of the study.

Measures of system use (e.g., numbers of questions) will be summarized descriptively and may be included as covariates in exploratory analyses to better understand adherence patterns; these analyses will be considered hypothesis-generating rather than confirmatory. All statistical tests will be two-tailed with a significance level of 0.05. Data analyses will be performed using IBM SPSS Statistics (version 24.0; SPSS Inc., Chicago, IL, United States) and Stata (version 14.0; StataCorp, College Station, TX, United States).

At 3, 6, and 9 months following the completion of the intervention, this study will conduct structured interviews to assess participants’ perception of the intervention as attractive, acceptable, easy to use, and satisfactory as a whole. These interviews will attempt to identify, in depth, the most prominent determinants of behavior change and engagement. Each interview should be approximately 30 min long to conduct and may be conducted over the telephone or face-to-face. Participant recruitment will take into account socio-demographic variables like residence, gender, age, education level, as well as stroke severity, to attain diverse representation. Content of interview will include participants’ own experience of the intervention, suggestions for improvement of AI-HEALS system, motivation to pursue self-management activities, as well as challenges that develop in the process. To attain richness as well as data breadth, interviews should be continued until thematic saturation has been achieved. All interviews should be audio-recorded, transcribed verbatim, as well as de-identification carried out on them. Data analysis should make use of the Theoretical Domains Framework (TDF) on NVivo 12 (Version 12, QSR International, Doncaster, Australia). Lastly, qualitative findings would be integrated with quantitative data to attain a richer understanding of the impact of the intervention as well as long-term effects on participants’ health behavior as well as outcomes.

## 3 Study management

The Data Monitoring Committee (DMC) would consist of no less than two members of the Ethics Committee of the Luoyang First People’s Hospital. None of the members would have any conflict of interest regarding the research. The members would monitor the progress of the research study periodically and inform the Ethics Committee. They would not be involved in the research. In the case of unapproved changes to the research, the members of the DMC would recommend the termination of the research. Although AI-HEALS is a behavioral and mobile health–based intervention, similar mHealth or AI-assisted self-management programs have generally demonstrated a strong safety profile, with few or no serious adverse events (AEs) reported. Expected adverse events (if any) are expected to be minimal and non-physiological in nature, such as frustration when using technological devices, increased psychological burden caused by frequent reminders, or inconvenience during system interaction. No direct biomedical risks are expected, as the intervention does not involve pharmacologic, device-based, or invasive procedures. In the case of any AEs and serious AEs occurring to the participant, it would be communicated to the principal investigators within 24 h, and the study would be paused for further check. In case of major technical failure of the AI system and serious problems that might pose risks to the participants, as well as any issues regarding the user interface, the study would be halted immediately. Repairs would be conducted by the technical team. After the system had been repaired and stability had been confirmed, the study could either be reframed and continued if it was safe and sound scientifically.

Prior to the initiation of the study, system-oriented training and intensive assessments aided by subject matter experts will be carried out to ensure that all team members acquire broad knowledge of study procedures and technical finer points. To ensure minimal loss to follow-up, each participant will be properly registered as well as provided with a unique identification code. Additionally, to ensure convergence of the intervention as well as control arms, all assessments will be carried out at standard time points utilizing validated questionnaires. To permit data accuracy, a double data entry as well as validation system shall be instituted, thus enhancing data processing quality. After data analysis, the research team shall continuously engage statistical experts to determine the best data analytical methods suitable for the data at hand. If data processing gives detected irregularity or anomaly, the original questionnaires shall immediately be reviewed to determine data accuracy. Only data validated as accurate shall constitute part of the final analysis. These rigorous procedures serve to safeguard the validity as well as reliability of study results.

Regular supervision of the research team by the principle investigator shall ensure strict adherence to study protocol and complete acquisition of study data. If it becomes evident that data collection or follow-up procedures have not been conducted according to established protocols, appropriate corrective actions may be taken, such as terminating the trial or modifying the randomization schedule. Data collection shall only continue following implementation of the needed changes. In the event that systemic or widespread errors are found to exist in questionnaire design, or data recording errors found on conduct of the study, the research team shall place on hold conduct of the trial temporarily for correction and redesign of the instrument. Redesign of the questionnaire shall be accompanied by re-administration of the questionnaire to participants to obtain data that accurately reflect the study goals as well as desired outcomes of the study. These quality control steps are necessary to ensure the scientific rigor of the trial as well as adding to the reliability as well as validity of the results of the study. All of the data shall be handled by a data manager of design for that role. Data entry functions shall have a trial manager to watch over them, deal with cloud storage, as well as resolve issues that involve data management as well as accessibility of that data. The trial manager shall also carry out routine audits to ensure that data obtained are accurate, consistent, as well as adherence to the study protocol.

Unblinding shall only be permissible on exceptional grounds. Situations may involve emergency medical situations where knowledge of group assignment becomes unavoidable, such as when there arises a serious adverse event or when emergency treatment must be established. Participants or their doctors may similarly ask for unblinding after the conduct of the trial for legitimate medical reasons. Unblinding must always be requested through a written application submitted by means of the principal investigator or through a member of a clinical oversight team. All such requests shall pass through a clinical adjudication panel for determination as to whether there are grounds for unblinding. If granted, the participant’s assignment shall be revealed through established procedures, balancing participant protection and confidentiality.

All changes that are suggested to the study protocol will first be considered and reviewed by the Steering Committee. After consensus, the changes that are suggested will be sent to the hospital’s Ethics Committee and Research Administration Office for approval. Following approval, an official statement of changes will be distributed to all persons involved, with electronic copies as needed. All changes to the protocol will be signed by the principal investigator upon notification by the trial coordinator. The paperwork will be stored among other documents of the trial. Following approval by the Steering Committee, Research Administration Office, and Ethics Committee, the trial coordinator will make corresponding changes to the clinical trial registration.

## 4 Dissemination plans

The research team will prepare a summary of the RCT findings and distribute it via email to participants who indicated interest in receiving study results on their informed consent forms. The findings will be disseminated through publication in peer-reviewed journals and presentations at national and international conferences. Additionally, the results will be made available to other hospitals upon request for dissemination among their clinical staff. Study data will also be shared upon reasonable request, in accordance with ethical and data-sharing guidelines (39, 40).

## 5 Discussion

Stroke is an acute disease that arises from the rupture or obstruction of brain vessels, which typically manifests as sudden neurological dysfunction such as limb numbness and disturbance of speech (4). Stroke has a high death incidence as well as long-standing disability (1, 2). According to the Global Burden of Disease Study 2019, stroke accounted for approximately 6.55 million deaths worldwide in that year, with about 101 million people living with the condition and a total of 143 million DALYs lost (2). While patients living through strokes of mild as well as moderate intensities document high rates of survival, they still register various intensities of functional dysfunction that may significantly impact their quality of lives dramatically (41–43). Additionally, provision of long-standing care as well as rehabilitation of such patients presents significant economic as well as psychological strains on patients’ relatives as well as on themselves, who may develop psychological as well as health conditions of their own (7, 8). Historically, the care and rehabilitation of stroke survivors have been delivered primarily through specialized health centers, a model that imposes substantial economic burdens and limits both the continuity and frequency of care. Physical rehabilitation has long prevailed over such interventions, which tends to forget the psychological well-being as well as the behavioral adjustment of patients. Correspondingly, there has long existed a need to devise new, patient-centered stroke interventions, as well as the development of LLMs presents a tenable alternative to meet such a need.

The AI-HEALS has the aim of enhancing stroke care through the use of artificial intelligence and mobile health technologies. It offers personalized health education as well as behavior change support to stroke patients and their families. Key system functions include knowledge-based question-answering services, health indicator monitoring, and personalized educational content and support tailored to the specific needs of stroke patients and their caregivers. Additionally, AI-HEALS enables care providers to monitor clients’ real-time system usage via a backend platform, facilitating the assessment of educational needs and the utilization of the intervention plan, thereby ensuring its effective implementation. Pilot studies have already indicated the tremendous promise of mobile technologies of health to deliver improved clinical outcomes when applied to stroke care. Through the support of large language models, AI-HEALS still promises to attain improved control of symptomatic improvement as well as improved recovery among patients that have mild to moderate stroke.

Based on this, this project will conduct a single-blind RCT to evaluate the effectiveness and acceptability of the AI-HEALS system in patients with mild to moderate stroke. Its primary objective will be to establish changes to patients’ blood glucose, blood pressure, and lipid profiles as a consequence of the intervention. Its secondary endpoints will include changes to perceived risk of stroke recurrence, physical health indicators such as hospital readmission rates, BMI, psychological and behavioral endpoints related to self-management of health plans, family involvement, as well as quality of life broadly. These endpoints will be examined using a combination of quantitative methods, such as standardized questionnaires and physiological measurements, and qualitative interviews. Due to the complex behavior and medical conditions involved when experiencing mild to moderate stroke, AI-HEALS has significant potentials of enhancing patient engagement as well as enhancing treatment guideline adherence.

In spite of the salutary possibilities of the planned intervention, there are certain limitations that must be recognized. First, the results may be limited in terms of generalizability as a consequence of the inclusion criterion, which aims at targeting a certain subgroup of the population that possesses specific clinical attributes. For instance, this research concentrates on patients suffering from mild to moderate stroke that are capable of utilizing digital health interventions, which may leave out patients that possess poor health literacy or have had little experience with technology. Secondly, while the research is anticipated to provide insightful knowledge on the effectiveness of the AI-HEALS system, there may be limitations in accounting for numerous external variables that impact patients’ perceptions about recurrence risk. Triggers from the environment, socioeconomic position, and accessibility to the services of delivering health, are all significant variables that may influence outcomes but cannot easily be controlled within real-world conditions. As a way of handling this, the study incorporates the demographic and the clinical covariates into the analyses of the study in an attempt to account for such influences, although there may still remain residual confounding that may impact the results. Finally, despite planning rigorous monitoring procedures designed to ensure data quality, there may still remain relevant issues such as participant dropouts as well as missing data that may still remain a threat to the validity of the results. The research team intends utilizing multiple imputations techniques as a way of overcoming such challenges; however, the possible bias that might arise from missing data must still remain carefully considered when interpreting the results.

## Footnotes

1
https://flageval.baai.ac.cn
